# Outcomes associated with azithromycin use among patients hospitalized with non-severe community-acquired pneumonia

**DOI:** 10.1017/ash.2025.218

**Published:** 2025-09-24

**Authors:** Ashwin Gupta, Emily Walzl, David Ratz, Jennifer Horowitz, Elizabeth McLaughlin, Tejal Gandhi, Lindsay Petty, Anurag Malani, David Paje, Preeti Misra, Scott Kaatz, Steven Bernstein, Mariam Younas, Scott Flanders, Valerie Vaughn

**Affiliations:** 1 University of Michgian/VA Ann Arbor Healthcare System; 2 VA Center for Clinical Management Research; 3 DIvision of Hospital Medicine, Michigan Medicine; 4 University of Michgian; 5 University of Michigan Medical School; 6 Michigan Medicine; 7 Trinity Health Michigan; 8 Trinity Health Livonia Hospital; 9 Henry Ford Hospital; 10 Michigan State University - College of Human Medicine, Hurley Medical Center; 11 University of Utah School of Medicine

## Abstract

**Introduction:** Community-acquired pneumonia (CAP) is the leading infectious cause for hospitalization. Guidelines recommend use of a macrolide antibiotic with a beta-lactam for coverage of atypical organisms; however, data supporting macrolide coverage disproportionately include patients with severe CAP. Debate remains regarding the benefit of macrolide coverage among patients hospitalized with non-severe CAP. **Methods:** We emulated a target trial to evaluate outcomes associated with azithromycin use among patients hospitalized with non-severe CAP between 7/2017 and 8/2024 across 69 hospitals in Michigan. Included patients had an ICD-10 discharge diagnosis code of pneumonia, >2 signs or symptoms of CAP, and radiographic findings. Patients with severe CAP, risk factors for multi-drug-resistant organisms, those not started on standard CAP therapy with a narrow-spectrum beta-lactam with or without azithromycin, or those initially receiving doxycycline were excluded. Time zero was the time of first antibiotic administration on encounter day 1 or 2. Groups receiving and not-receiving azithromycin were balanced using inverse probability of treatment weighting (IPTW) assessed using standardized mean differences (SMD). The primary outcome was time to clinical stability. Secondary outcomes included intensive care unit (ICU) transfer, 30-day rehospitalization, 30-day mortality, and protocol deviation (i.e., azithromycin initiation after time zero [no-azithromycin group], patients receiving <5 days of azithromycin [azithromycin group]). We used the Cox model and multivariable Poisson regression for time-to-event and binary outcomes, respectively. Based on point prevalence of outcomes within our cohort, we were well powered to detect the demonstrated relative differences in all outcomes. **Results:** Of the 59,698 patients meeting criteria for pneumonia, 19,108 patients were included in the final post-exclusion cohort. Of these, 93.7% (17,904/19,108) received azithromycin on day 1 or 2 (median antibiotic duration 4.0 days [IQR 3,5]), while 6.3% (1,204/19,108) did not. After IPTW, groups receiving and not-receiving azithromycin were well balanced (SMDs <0.1). After adjustment, median time to clinical stability did not differ between the azithromycin and no-azithromycin groups (3 vs 3 days; HR 1.01 [95% confidence interval 0.97–1.14], p=0.74), nor did rate of ICU transfer (0.9% vs 1.3%; HR 0.90 [0.51–1.62], p=0.73). Patients receiving azithromycin had lower rates of 30-day rehospitalization (10.8% vs 15.3%, HR 0.69 [0.58–0.82], p<0.001) and 30-day mortality (2.3% vs. 4.0%; HR 0.70 [0.50–0.93), p=0.03). Protocol deviation occurred more commonly in those initially receiving azithromycin (56.5% vs 11.1%; HR 1.58 [1.32–1.82], p<0.001). **Conclusions:** Addition of azithromycin to beta-lactam therapy in patients hospitalized with CAP did not influence short-term outcomes but may reduce 30-day rehospitalization and mortality.

